# The production of on-line dialysis water for extracorporeal dialysis: proposals for an increased safety upgrade: a viewpoint

**DOI:** 10.1007/s40620-019-00667-2

**Published:** 2019-11-12

**Authors:** Piergiorgio Bolasco

**Affiliations:** Regional Public Institution, Cagliari, Italy

**Keywords:** On-line dialysis fluid water, On-line quality of water, On-line water on-line upgrading, On-line water hemodialysis safety, Double-osmosis, On-line hemodialys plants

## Abstract

**Introduction:**

At the start of the 2000s, the progressive diffusion of high-flux extracorporeal dialysis and membranes saw an increased use of high infusion volumes injected into the patient’s blood circuit following the advent of on-line water production plants.

**Methodology:**

Our 15-year experience with on-line extracorporeal methodologies using very high infusion volumes has led to the detection of errors and weaknesses, thus allowing us to correct and provide for the implementation of appropriate technology in dialysis water production plants with the aim of ensuring a higher chemical-physical, bacteriological and endotoxin quality. The initial procedures had already been outlined in the 2005 Italian Guidelines, although still today Health Technicians and Nephrologists operating in the field are unable to take on board specific integrations for on-line methods due to a lack of upgrading of documentation in both European and non-European Guidelines.

**Results:**

After more than 17 years’ experience, and in view of the technological implementations developed since 2005, we wish to put forward a series of suggestions in an attempt to improve the safety of on-line water, with uses ranging from drinking water, pre-treatment, osmosis, distribution circuit, hemodialysis monitors up to the most recent update of microbiological cultures.

**Discussion:**

Additional, more stringent measures are required to prevent the occurrence of acute accidents during dialysis sessions and to reduce chronic inflammation-oxidation deriving from the use of not totally ultra-pure/sterile dialysis fluids.

**Conclusion:**

Our point of view based on our long-standing experience, the proposals made relate to procedures to be applied in technological maintenance, which the consultant nephrologist and other relevant personnel such as microbiologists, biologists, and technical operators should adhere to rigorously to ensure that the production of dialysis water on-line is viewed on a par with a pharmacological administration.

## Introduction

The start of the second millennium witnessed an increased interest and marked escalation throughout Italian and European nephrology units in use of on-line extracorporeal dialysis methods (OL-HD). Indeed, for more than 15 years, high volume exchange techniques such as post-dilution hemodiafiltration [1–4], pre-dilution hemodiafiltration [5], mixed hemodiafiltration [6, 7], and mid-dilution hemodiafiltration [8] have been applied due to the potential clinical benefits of these methods and to their higher depuration efficiency in removing high molecular weight toxins. The use of these methods implies direct infusion into the patient’s blood stream of large quantities of dialysis fluid produced on-line, at times corresponding to the total amount of body water [9], with the aim of guaranteeing adequate depuration of a wide range of toxins of different molecular weights (MW) from urea to those with a high MW bound to plasma proteins, which require a higher infusion flow to achieve increased removal [10–13]. Following the evolution of dialysis methods, application of this type of high-flux convective extracorporeal technique has implied an increased need to ensure the quality and safety of dialysis water produced on-line by improving a series of procedures and systems, in particular double-osmosis treatment plants (DRO) [14, 15]. The latter produces water with a purity comparable to that prescribed for ultrapure, non-pyrogenic water by the European Medicines Agency, and is indicated for use either as an intravenous infusate or dialysis fluid in extracorporeal dialysis techniques. The on-line production of dialysis water using the tap water supply has revolutionised the costs deriving from use of copious litres of sterile infusions, whilst at the same time eliminating the need to store containers of non-sterile basic and acid dialysate concentrates that frequently results in uncontrolled bacterial growth and development of endotoxins caused at times by long periods of storage in warehouses not suited for the purpose. In Europe, no reliable data are available with regard to the use of DRO, Single Reverse Osmosis (SRO) and on- line extracorporeal dialysis methods. Indeed, although not updated for recent years, data available for Europe are as follows: 13.8% of hemodialysis patents have been treated using on-line hemodiafiltration methods (OL-HDF) throughout Europe [16], 30% in France in 2016 [17], and 79% in Japan in 2016 [5]; the technology is used to a lesser extent in Asia and Canada and rarely in the United States. This increasing interest for OL-HD worldwide strengthens the lynchpin of this paper, represented by the Guidelines relating to the Treatment of Dialysis Water published by the Italian Society of Nephrology (SINGL), which still remain a reference point today [18]. The Italian Guidelines, drafted and published in 2005, also addressed the management of on-line extracorporeal dialysis methods (OL-HD) although, at the time the guidelines were drafted, on-line extracorporeal techniques were still in their infancy and adopted by very few dialysis units. However, 14 years later the Italian Guidelines are still regularly consulted by thousands of nephrologists worldwide. This continuing interest is likely due to the fact that in Europe, the latest update on Dialysis Water Guidelines was conducted in January 2019 by the Renal Association and the Association of Renal Technologists in the United Kingdom [19]. In no other international guidelines, have chapters relating to upgrades, new safety limits and/or new advice been specifically devoted to OL-HD. Accordingly, the present suggestions and proposals for optimization and update of the guidelines are the result of the collection of a vast body of findings from microbiological, bacterial and endotoxin assays, which in our experience have promoted a gradual evolution over a period of 17 years [15, 20], culminating in the advent of safer clinical strategies aimed almost exclusively at optimizing the characteristics of dialysis fluid for use in OL-HD. Indeed, although only sporadically, severe incidents may still occur today in the context of hemodialysis in spite of the application of DRO; these incidents are manifested in the presence of an excessive complacency in use of the equipment, and a lack of training in the correct performing of maintenance work and disinfection of equipment [21–25]. It should also be considered that today the hemodialysis patient population is represented by more vulnerable hemodialysis groups such as the elderly and/or patients affected by major comorbidities. In this document, we therefore propose to provide in-depth details of all relevant procedures for the entire range of professionals involved in the management of dialysis water: Nephrologists, Public Health Clinicians, Microbiologists, Biologists, Chemists, maintenance Staff and Lab Technicians. Numerous dialysis centres continue to not use the highest quality methodologies; however, this outcome could be progressively obtained, step by step, by fostering an increased synergy between the professional figures implicated in these crucial, but delicate, procedures. The aim of this paper is not to focus on the acute or chronic clinical consequences of the thousands of litres of dialysis water that are infused into, or come into contact with, our patients every year, nor to underline the inflammatory and oxidative reactions manifested as a result of contact with less than ultrapure dialysis fluid, or even to focus on the clinical benefits experienced by patients receiving on-line extracorporeal technologies thanks to the replacement of their body water with tens of litres of dialysate. Indeed, in line with more than two decades of experience of hemofiltration in online predilution, we feel compelled to put forward a series of recommendations to be implemented alongside those provided in the Italian Guidelines [18], and to reinforce the concept among nephrologists that water for HD-OL should be considered on a par with other pharmacological products administered into the bloodstream of hemodialysis patients.

## New trends and integrations in the production of water for use in on-line extracorporeal dialysis technologies

### Tap water supply

#### Knowledge premise

The Hospital Health Director is responsible for the quality of water downstream of the property water meter, whilst responsibility for plant design and construction lies with the construction companies. A fully comprehensive maintenance contract should be underwritten with the contractor, comprising a guaranteed accuracy of control sampling; in addition, wherever possible, the main contractor should be asked to appoint a fully certified environmental hygiene laboratory. As manager of this facility, the nephrologist carries out a role of fundamental importance and responsibility, particularly in view of the complexity of the issue and the need for complex technical knowledge. The nephrologist will need to acquire detailed knowledge of the characteristics both of the water supply and water used for medical purposes, including dialysis water [26, 27], specifically to enable him to effectively review maintenance reports and interpret the findings of physicochemical, bacteriological and endotoxin assays and set up an electronic storage system for the filing of all reports. This concept is also reiterated in the English Dialysis Guidelines under point 1.2 [14, 19]. Currently, in the majority of cases, a Hospital Health Director faced with harm to or death of a patient due to deviations in dialysis water features, may refuse to accept responsibility by stating he is a “technician lacking the required competency”, thus implying the need for a Competent Technician to deal with the issue directly, i.e. the Head nephrologist, with the statement provided in the SINGL affirming “the complexity of the issue and the technical knowledge required are such that the nephrologist should not be expected to assume liability for issues beyond the remit of his position” then continuing “however, the Unit Director should not be deemed responsible for the quality of water administered to his patients, clearly no longer being acceptable. Indeed, to avoid this event, in the light of this situation, a close collaboration should be established between the relevant health authorities, competent technicians, hospital technicians, public health officials, microbiologists and nephrologists, the latter of which should coordinate, monitor and ensure the implementation of safer, updated advanced technologies. Additionally, it should come as no surprise that numerous nephrologists completely overlook the periodic monitoring, either directly or through the relevant authorities, of the chemical-bacteriological data relating to the water supply, subject to a wide range of variations based on seasonality, climate, rainfall, and chemical pollution of municipal and rural water tables, recently exacerbated by the use both in Italy and elsewhere of fertilizers enriched with halogenated hydrocarbons [28]. The lack of communication between Regional Environmental Agencies, Municipalities and the local Health Authorities is considerably more widespread than envisaged, thus implying the need to set up a formal agreement between the water supply company, those responsible for monitoring and control and the company supplying dialysis water to patients.

## Technological in-depth

**Fig. 1:**
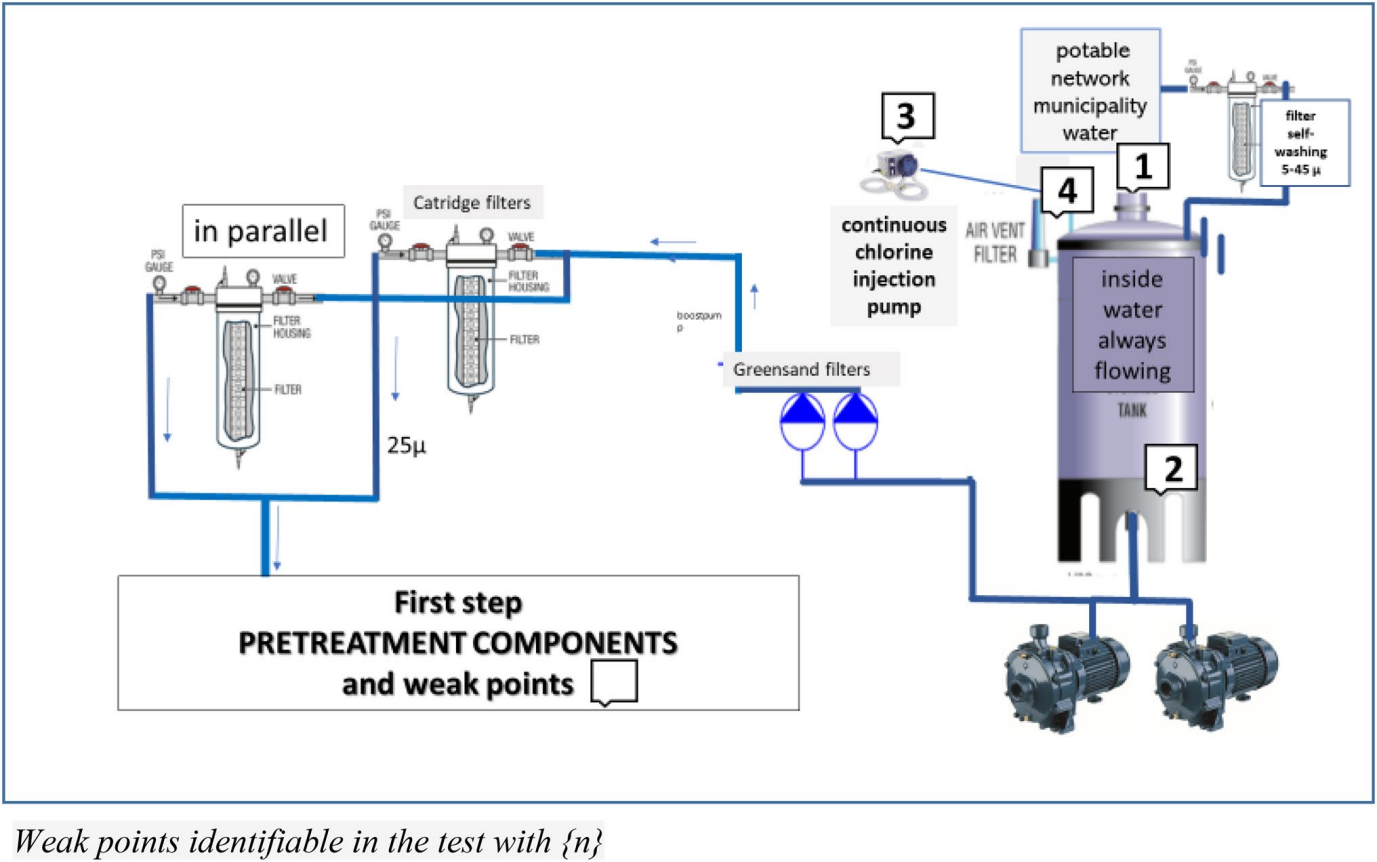
Pre-treatment first step and weak points

**Fig. 2:**
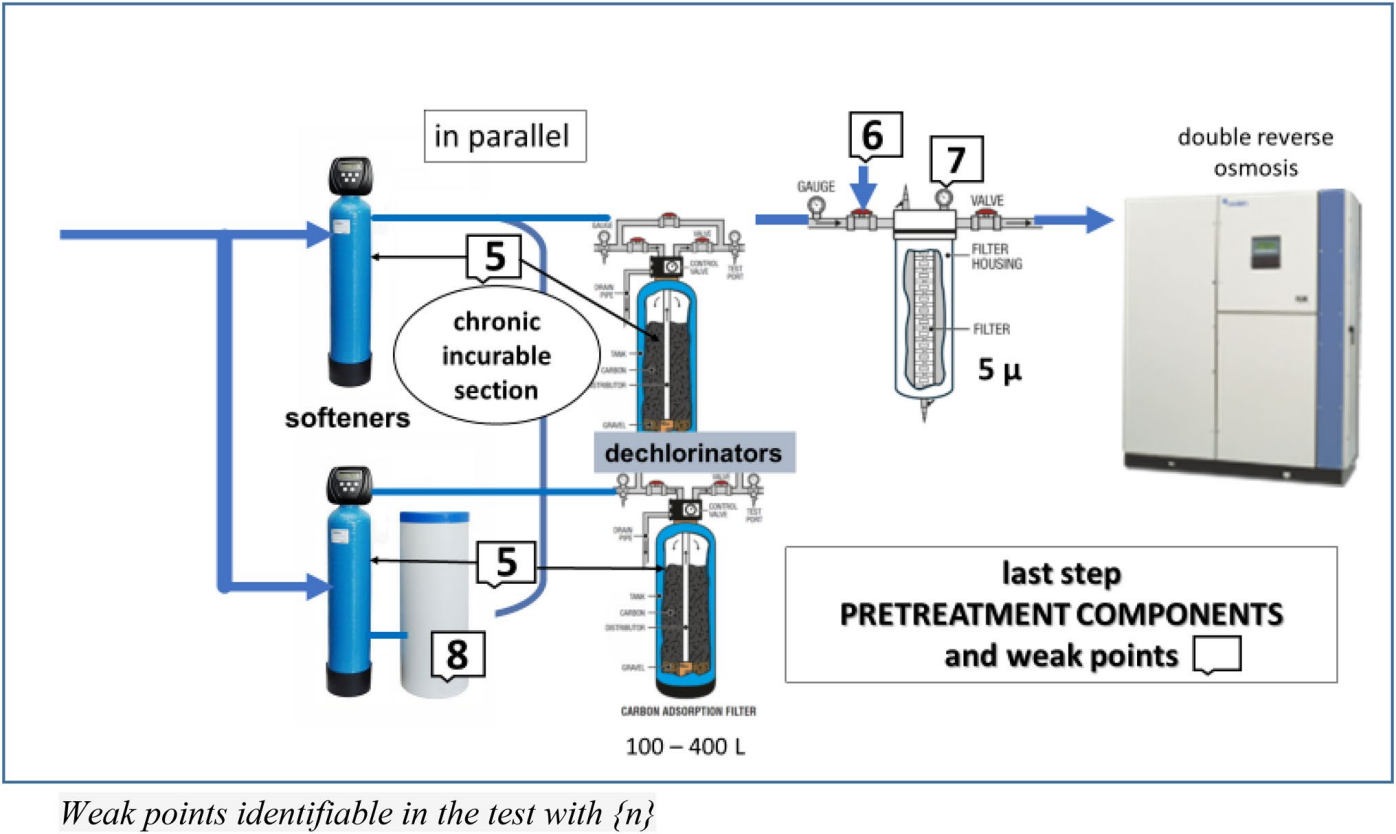
Pre-treatment last step and weak points

Figures 1 and 2 illustrate the range of components implicated in the set-up of an ideal or optimal water pre-treatment plant in line with the current state-of-the-art. The ‘weak’ points and/or those requiring particular care are numbered in the diagram.

### Pre-treatment

With regard to pre-treatment, there is little to be added to the previous SINGL, with the possible exception of the fact that until a more appropriate technological solution is identified, pre-treatment should be deemed an incurable chronically ill patient. The least curable part is represented by the softener unit, a fertile breeding ground for all types of microorganisms, fungi and spores; it is well-known the impossibility of disinfecting but it is indispensable cleaning dechlorinator and softeners bed from organic and mineral water residues by overnight automatic backwashing on a daily basis to avoid a decrease in effectiveness and thus prolong the life of RO membranes. Samples collected downstream of the dechlorinator have revealed growth of Pseudomonas Aeruginosa, total coliform and other bacterial species [29, 30]. Also, in our experience we detected the presence of Pseudomonas Aeruginosa (from 90 to 4000 CFU/mL) (personal observation during follow-up periodic time). The softener and dechlorinator beds, the latter containing active carbon, should be sized based on the water demand needed to meet the requirements of the maximum number of technical beds (from 100 to 400 L), and in line with the hardness of incoming water. A series of ad-hoc options are available for use in the sizing of dechlorinators. However, from pre-treatment onwards, several weak points can be identified, as reported by the numerical references indicated in Figs. 1 and 2. A filter (generally comprised of a fine polypropylene fibre mesh) ranging in porosity from 5 to 45 µ aimed at retaining water impurities should be placed at the point of water inlet into the production unit from which water if directed towards the water tanks {1}.This system is particularly effective if the maintenance contractor equips the filter with an automatic washing function in order to avoid frequent substitutions. The SINGL are relatively exhaustive on the matter of the storage tank. The tank walls should be dark to avoid the infiltration of light and the tank should not be placed in direct sunlight or exposed to heat to avoid the possible undesired growth of extensive algal colonies [31]. An additional recommendation for the maintenance contract relates to the emptying and disinfection of the tank, even in the case of an underground “communal” tank containing thousands of cubic metres of tap water. The water storage tank should be “dynamic” and not occupy a dead space in the treatment plant, i.e. the inlet and discharge of water should flow constantly {2}. Moreover, the tank(s) represent the ideal point for addition of chlorine to water influx using a high precision, possibly not peristaltic, injection dosing pump, to overcome potential pressure issues following introduction of chlorine {3}. The optimal dosing of chlorinated water is 0.5 ppm, given that the drinking water supply is already chlorinated, and thus the effectiveness of dechlorinators will be extended. Direct communication of the tank with the external environment should be ensured through a device equipped with an air filter {4}. Lastly, with regard to the quartzite filters downstream of the delivery pump, periodic maintenance is required, including yearly replacement of softener and dechlorinator beds and active carbons {5}, a monthly check-up of chlorine and/or derivates downstream of the softeners (the presence of trihalomethanes is indicative of exhausted active carbons) {6}, and a 25µ micro-filtration pre-softener and 5µ post-dechlorination/pre-osmosis {7}, taking into account the recent introduction on the market of automated self-cleaning water filters with a stainless steel wire mesh that cyclically self-cleans using an integrated brush. The use of high-quality rock salt tablets for softeners is recommended {8}.

### Double-osmosis

Use of a double pass osmosis system is mandatory when performing on-line hemodialysis. The SINGL describe in detail the use and maintenance of this technology, with particular focus on preventing alterations to or degradation of the polyamide membranes. However, due to unforeseeable physicochemical factors or poor disinfection, microfractures/microlesions may be manifested on the thin polyamide sheets, thus allowing a series of micro-organisms to pass through. Accordingly, the guidelines recommend periodic monitoring of dialysis water quality in order to identify potential procedural deviations.

Recent innovations in osmosis systems have focused on a renewed interest in heat disinfection, which has proven to be particularly effective [32], with operational benefits afforded by automation of the disinfection process. Indeed, new modules have recently been proposed for a double-osmosis plant with automated integrated heat disinfection and scope for extension and personalisation to rationalise the demand for osmotic water based on the number of dialysis stations. This results in saving of electric power and water, a prolonged life for osmosis membranes with reduced bacterial biofilm on the piping ring circuit. The system is equipped with a Hot Water Tank that supplies water at a temperature of 85° C overnight (approx. 10 h) to 1-8 osmosis modules as required. These osmosis modules, already available on the market, (CPW800-Baxter^®^), provide both chemical and heat disinfection for up to 12–32 dialysis stations, extending to more than 40 dialysis stations when applying integrated automated heat disinfection solely to the ring/dialysis monitor connector. The novelty of this type of osmosis plant is represented by an optimized use of heat for disinfection based on maintaining a constant temperature over a given period to guarantee a fungicidal, bactericidal and virucidal action. The latter is achieved through calculation and measurement of the A_0_ parameter, thus determining the capacity of the heat disinfection cycle, promoting the inactivation of micro-organisms [33–35].

This represents a unit of time at a specific temperature A_0_ = t × 10^**(**T−80)/10^ (where *t* indicates the maintenance time of disinfection temperature in seconds, and T the disinfection temperature in °C). Reference values for A_0_ are provided for in the EN ISO 15883-1:2006 standard. To conclude this chapter, a brief mention should be made of the ambient temperature required in the building housing the treatment plant: the area should be maintained at a temperature of < 25 °C to enhance plant yield and prevent development of non-mesophilic pathogenic micro-organisms deriving from water heated on passing through tubes and/or tanks exposed to sunlight or heat.

### Distribution ring

**Fig. 3:**
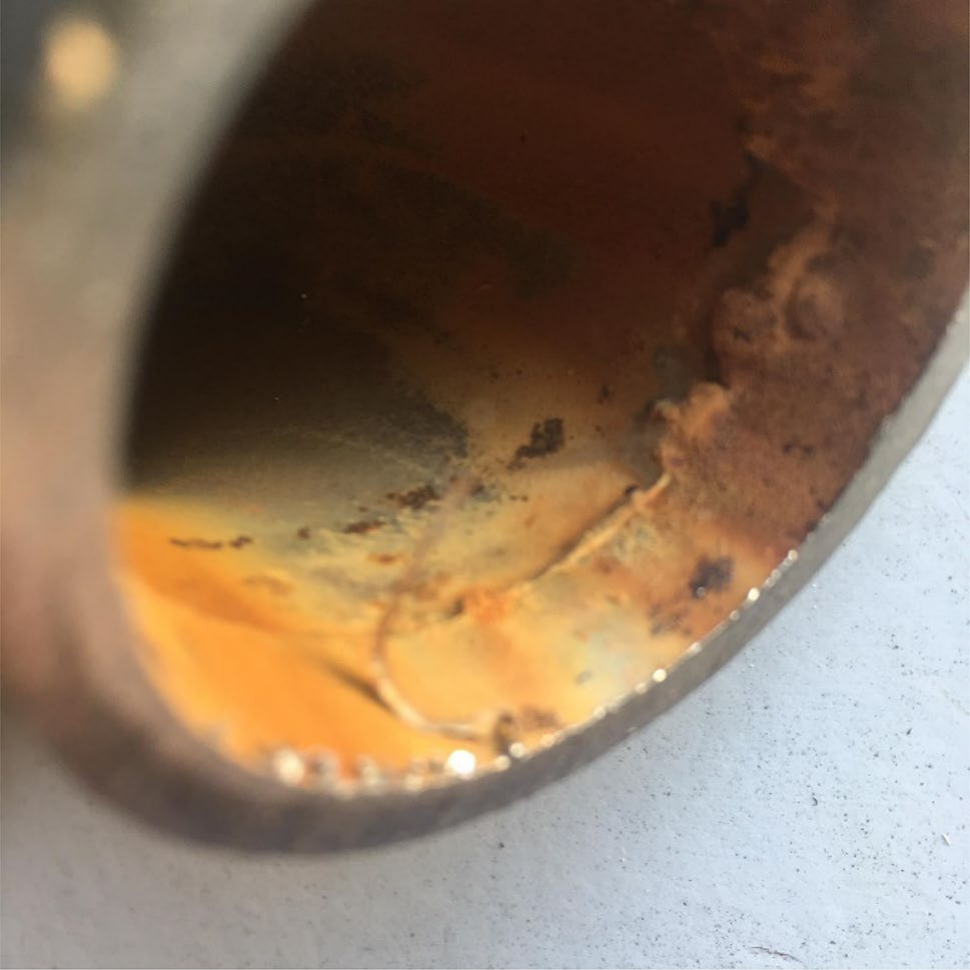
Welding section after six years of use of inox AISI 316L piping ring. It is possible to note fusion defects by hot weld cracking. This problem was found in three dialysis centers after the removal of the stainless steel and its replacement with PEX

Both the type of closed loop circuit recirculated to the osmosis unit and the materials used: stainless steel (INOX), cross-linked polyethylene (PEX) and polyvinylidene difluoride (PVDF) are well established. When opting to use INOX for both the ring and connector valves to the monitor, a smoother type of steel such as AISI standard 316L or higher is recommended. Likely due to the less widespread use of this type of system as a result of the high costs involved, no reports are present in literature on the long-term outcome of use of INOX with specific regard to the antibacterial effect produced. Stainless steel circuits are undeniably long-lasting, thus contributing towards amortization of costs; however, due to the impossibility of obtaining unwelded piping, electro-chemical methods should be adopted. Tungsten Inert Gas (TIG) arc welding is characterised by use a tungsten electrode which, protected by an inert shielding gas such as Argon or Helium, welds the two parts. This type of welding requires a high precision performance by the operator and may result in the production of *hot weld cracking* (fusion defects) caused by a lack of cleanliness of the metal edges to be welded or the presence of contaminants. There is indeed no certainty that the welded segments will not release rust or shards that may result in the development of bacterial or mineral biofilm, particularly following thermal disinfection of the ring and potential thermal oxidation. In the Authors’ experience, inspection of the INOX piping may reveal a coarse surface and small deformities in the connector (personal observation), as displayed in in Fig. 3. An important turning point was represented by the advent of PEX; it is however crucial that the entire ring is comprised of a single PEX tube with no welding, which would inevitably result in shards and the potential development of bacterial biofilm. Moreover, in our experience the number of bacterial CFUs observed when using PEX tubing are significantly lower than those detected for the previously described INOX rings [14]. PEX tolerates temperatures of up to 90 °C, and, similar to other piping materials, should feature a diameter not exceeding 1.5 cm; the smaller diameter, higher velocity and shear stress of osmotized water along the inner walls of the pipes prevents the formation of air pockets facilitating development of bacterial biofilms, which are frequently not fully removed by thermal disinfection. Indeed, in the Authors’ opinion, although effective, thermal disinfection should be alternated, at least on a monthly basis, with chemical disinfection, preferably using peracetic acid, to promptly remove organic or micro-mineral biofilms that create a breeding ground for bacteria, as well as to prevent release of their cellular fragments, particularly muramylpeptides and polysaccharides. The efficacy and safety of these options are described in the SINGL.

### Ring-monitor connections between piping ring and hemodialysis monitors

This topic is not addressed in the SINGL and continues to be overlooked in numerous dialysis units. The issue is however of fundamental importance and capable of neutralizing the outcome of even the most efficient disinfection procedures. The elimination of mesh tubes as a ring-monitor connector is strongly recommended. In a multi-centre study conducted by the authors, cultures grown from swabs taken at the opening of the mesh tube revealed a bacterial load exceeding 10,000 CFU/cm^2^ [20], comprised of mesophiles and pathogens such as Pseudomonas Aeruginosa. Accordingly, the well-established flexible PEX tubing should be used at the shortest length possible and undergo scheduled periodic thermal/chemical disinfection. Junction points linking the tubing to the rings and monitors should be manufactured from high quality INOX.

### Reuse of waste water

Systems focusing on the reuse and recirculation of double-osmosis waste waters, which would contribute towards further reducing water usage, are currently undergoing investigation. Conversely, with regard to the discharge of waste waters from dialysis units, a sentence issued by the Italian Supreme Court of Cassation states that due to the presence of micro-pollutants, waste waters originating from a dialysis unit should be classified as industrial rather than domestic waste waters (Supreme Penal Court of Cassation Section 3° at 31/08/2016 hearing dated 10/05/2016, sentence n.35850). Discharge of these waste waters therefore is subject to prior authorization.

### Filtration in dialysis machines

A range of different dialysis machines is present on the market, with those compatible with the on-line preparation of dialysis fluid featuring at least two filtration phases. Some manufacturers apply two filtration phases only in the case of on-line technologies whilst others use two phases for all methods. Some companies, with the aim of further enhancing water quality, include an additional filtration phase at the point of water inlet to the dialysis monitor. This third stage of ultrafiltration could be considered a further safety control for water quality thanks to the presence of three filtration phases [36–38],

## Water quality assays and actions in on-line extracorporeal technologies

Prior to providing recommendations relating to the management of and scheduled/non-scheduled maintenance work for OL-HD, in addition to new suggestions for chemical, biological and endotoxin control assays, we should like to review the table of microbial and endotoxin loads illustrated in the SINGL, with particular focus on the need to adhere to more stringent levels of tolerability (Table 1) also compared to relatively recent publications [39]. The reference values listed in the SINGL for chemical and physical characteristics remain valid. The control sample should be obtained at the point of water inlet to the dialysis monitor by disconnecting the ring–monitor connector.

**Table 1:** Microbiological-endotoxins ideal control limits for on-line extracorporeal dialysis treatment

	Drinking water^a^	Water for on-line dialysate and infusion	Frequency
Bacteria CFU/mL, 22 °C	< 100	0	Every 2 months
Bacteria CFU/ml, 35–37 °C	< 20	0	Every 2 months
Molds and yestes/mL	–	0	Every 2 months
Endotoxins EU/m	< 0.25	< 0.01	Every 2 months

Published in the Ordinary Supplement to the Official Italian Gazette 3 March 2001 n. 52

^a^Text updated to 29 August 2017 from Legislative Decree 2 February 2001 n. 31 of Implementation of Directive 98/83/EC relating to the quality of water intended for human consumption

### Dialysis unit monitoring procedures and specialist lab assay

Technical operators employed by the contractor and/or public health departments (e.g.: Regional Environmental Agencies), implicated in monitoring processes, maintenance works and required interventions in a dialysis unit, should be specially trained. We therefore deemed it opportune to integrate the at times seemingly banal provisions established by the SINGL, which may prove fundamental in interpreting the results and outcomes of these procedures with the aim of ensuring patient safety. A written report of all scheduled and unscheduled maintenance work undertaken should be provided to the Clinician in charge of the Unit. It may be useful, on completion of work, for the contractor or Public Health Department to draft a comprehensive report attesting the safety of all electrical, physical and chemical parameters, providing a description of the work carried out and a detailed report of the bacteriological and endotoxin data collected.

### Points and methods of water sampling

As provided for by item 3.1.2 of SINGL [18], chemical purity should be assessed at least at the following two points: (1) mains water at the entrance to the system wastewater treatment, and (2) water treated downstream of the osmosis plant. Indeed, the microbiological charge should be evaluated every 2 months for each dialysis monitor (Table IV.1), and in the presence of bacterial load and/or endotoxin concentration above the recommended limits, bacteriological investigations should be extended to multiple points in the plant: (1) post water softener, (2) post dechlorinators, (3) post-osmosis downstream of the ring connection pipes distribution-monitor (particularly in the last tap ring).

In our experience, samples should be collected from the following points: (1) mains water at the entrance to the dialysis station (physical–chemical-bacteriological testing), (2) pre-osmosis (physical–chemical testing aimed at highlighting the presence of chlorites), (3) one to three randomized samples to be collected from different points based on the number of dialysis stations because in our opinion sampling at each dialysis monitor would be useless and very expensive; these samplings should be carried out at the point of water inlet to the monitor-end of the connecting line to dialysis stations (bacteriological-endotoxin testing), (4) at termination of the ring and return to osmosis (bacteriological-endotoxin testing). Samples should be collected over the shortest time possible (a few minutes) in purpose-designed sterile canisters of at least 100 mL capacity, avoiding any form of contact and using a mask to cover mouth and nose. The stainless-steel terminal/point of inlet to the monitor should be accurately sanitized using sodium hypochlorite 10% and/or other sterilising solution, taking care not to overuse the product and to prevent drippage, as these may result in an underestimation of the actual microbial load. Waste water samples should be collected in borosilicate glass bottles or, preferably, disposable polyethylene bottles.

### Transportation of samples

On completion of sample collection canisters should be placed in a thermal container, such as a battery-operated portable fridge equipped with a thermocouple to ensure a constant temperature of below 10 °C (optimum 2–8 °C), up until delivery to the testing lab within a time frame of 2–4 h after sampling. Care should be taken not to overturn the samples. These precautions will prevent bacterial growth that may result in overestimation of actual sample load [40].

### Specific microbiology applications and determination of endotoxins

As mentioned previously, events manifested even rarely following bacteriological contamination of waters by mesophiles and fungi, but also by pathogenic micro-organisms that live and breed at temperatures close to that of the human body, must be prevented. For this reason, in recent years, the tendering of maintenance work comprising microbiological and endotoxin testing has enabled the cultivation of micro-organisms potentially capable of colonising pre-treatment plants; the presence of these micro-organisms cannot be ruled out as they may gain access through fissures in osmosis membranes caused by pressure surges or chemical and physical alterations. This tender document may be of use to microbiologists working in a specialist Public Health lab and contribute towards promoting an improved synergy with the nephrologist in charge of the unit. In the Authors’ opinion, microbiological testing procedures should not only assess the presence of environmental mesophiles and mycetes but should also address the issue of detecting specific pathogens. Accordingly, further details and updates relating to the procedures to be implemented are provided for by the new UNI EN ISO standards. For each of the microbiological procedures we referred to ISO 13959:2009 and (UNI EN ISO 6222:2001) [41] for the different bacteria with the aim of identifying and implementing further improvements and applying the specific Italian methodologies described in the sections below. We also considered procedures applied by other authors [42–46]. The choice of parameters to be tested depends in particular on the water supply system. In the majority of cases drinking water deriving from spring waters and waters from artificial basins is subject to seasonal variations, in addition to the fact that a fall in pressure loads the “colander” nets of the municipalities; this may potentially result in bacterial growth if the underground water tables are polluted by strata of black waters, thus culminating in poor water quality. Given the precariousness of the sample, also in view of the low concentration of free residual chlorine detected (0.01–0.02 mg/L) and the use for which it was intended, it was deemed important to identify indicators of faecal contamination in countries where surface water is used in the production of drinking water. In almost all cases, approximately 100–250 mL of the sample collected should be filtered through a 47 mm cellulose ester membrane with filtration characteristics corresponding to a nominal pore size of 0.45 μm.

## Bacteriological and endotoxins updating

With regard to the methodologies related to bacteriological cultures, particularly those focused on pathogenic bacteria that grow at the temperature of the human body, we should like to briefly mention a few updates. Total Coliform: the UNI EN ISO 9308-1:2002 reference has been updated to UNI EN ISO 9308-1:2014 [47]. *Enterococci*: UNI EN ISO 7899-2: 2003 remains valid [48]. *Pseudomonas aeruginosa*: the UNI EN ISO 12780:2002 reference has been updated to UNI EN ISO 12666:2008 [49]. *Clostridium perfringens*: the most recently updated procedures can be found in ISO 41001:2018. Mycetes and Mesophiles counting colonies: the reference cited in UNI EN ISO 6222:2001 remains valid [50].

### Endotoxin procedures

Almost all dialysis units perform Lymulus Amebocyte Lysate testing using either portable equipment or in lab-based assays using equipment periodically calibrated for this purpose; a series of technologies may be applied to perform the test, the results of which are available within a time frame of approx. 60 min. The methodologies applied comprise the gel-clot LAL assay, a basic qualitative method suited to low-volume laboratories, involving use of a kinetic turbidimetric reagent that performs using a single product both kinetic and gel-clot analysis with accelerated reaction times and no pre-incubation, coupled with a microplate reader equipped with endotoxin-measuring software, or the kinetic Chromogenic LAL Test (Charles River^®^) [51]. These products are all FDA-approved, with test sensitivity gauged to detect 0.001 EU/mL. Additional products are available for use in determining endotoxin levels by detection of peptoglycans and short fragments of bacterial DNA [37], however, both the quality/price ratio and, in particular, sensitivity to endotoxins, which should be no less than < 0.003 EU/mL, should be taken into account.

## Conclusions and summary

In view of the lack of detailed Guidelines for on-line extracorporeal dialysis methods, we have cited the most recent bibliographic references relating to the treatment and quality of dialysis water.^1^,^2^,^3^,^4^,^5^,^6^,^7^,^8^,^9^,^10^,^11^,^12^,^13^,^14^,^15^,^16^ However, we also wish to underline the high relevance and ongoing validity of the provisions established in the 2005 SINGL [18]; indeed, we herewith put forward a series of integrations required in light of the advent of on-line extracorporeal dialysis technologies:

1. Double-osmosis, configuration of the circuit loop, the use of appropriate materials for the loop, are all fundamental requisites, which should be strictly adhered to when using on-line technologies.
2. Dialysis monitor: CE-certified equipment for on-line technologies envisaging use of at least two ultrafilters to provide 100% bacterial retention.
3. The ultrafilters and hydraulic circuit of the monitor should undergo regular disinfection schedules (chemical and/or thermal) with proven efficiency for on-line technologies; the softener beds, dechlorinator beds and ultrafilters should be replaced in line with *manufacturer* indications.
4. Concentrates: basic or acid solutions or sterile, ultrapure and/or non-pyrogenic powders should be used to dilute osmotized water having the parameters indicated in the document referred to in Table 1.
5. Monitoring and bacteriological limits: limits established for traditional dialysis by the SINGL as indicated in Table 1 should be adhered to; our suggestion is these should be applied with particular rigour for on-line technologies.
6. Record of traceability: if this method is to be used, the recording of procedures is of fundamental importance.
7. Mandatory staff training programmes should be envisaged for clinicians, nursing staff, microbiologists, chemists and all technicians employed by maintenance firms with the aim of raising awareness of the requirements of on-line technologies.
8. The prescription of on-line technologies for patients receiving home hemodialysis is strongly discouraged as in the absence of ISO certification or scientific validation, the portable treatment devices used are not capable of complying with the bacteriological-endotoxin parameters reported in Table 1.
9. Machine filters, osmosis membranes, ultrafilters and dialysis membranes are manufactured using extremely high standard industrial processes and quality controls; however, it should be taken into account that, although rarely, at any point from the manufacturing process to the dialysis session breaches may occur, from the tap water supply to the unit to the administration of intravenous infusions during high-convection extracorporeal technologies; clinicians should never disregard the fact that they are administering pharmacological solutions.

## Footnotes

1 American National Standards Institute, ANSI/AAMI RD62; 2001 Water treatment equipment for hemodialysis applications.

2 BS EN ISO 13959: 2015: Water for haemodialysis and related therapies.

3 BS EN ISO 11663: 2015: Quality of dialysis fluid for haemodialysis and related therapies.

4 BS EN ISO 13958: 2015: Concentrates for haemodialysis and related therapies.

5 BS EN ISO 26722: 2015: Water treatment equipment for haemodialysis and related therapies.

6 BS EN ISO 23500: 2015: Guidance for the preparation and quality management of fluids for haemodialysis and related therapies.

7 NHMRC, NRMMC (2011) Australian Drinking Water Guidelines Paper 6 National Water Quality Management Strategy. Version 3.0. Updated December 2014. National Health and Medical Research Council, National Resource Management Ministerial Council, Commonwealth of Australia, Canberra.

8 Guideline for dialysate quality of Spanish Society of Nephrology (second edition, 2015).

9 American National Standards Institute, ANSI/AAMI RD62; Water treatment equipment for hemodialysis applications—2001.

10 Guidelines for Environmental Infection Control in Health-Care Facilities; Recommendations of Practices Advisory Committee (HICPAC) CDC and the Healthcare Infection Control; U.S. Department of Health and Human Services Centers for Disease Control and Prevention (CDC): Atlanta, GA, USA, 2003.

11 Reference Analytical Methods for Drinkable Water Second Law by Decree 1; Chemical—31/2005.

12 Reports ISTISAN07/5; Health Ministerial: Rome, Italy, 2007.

13 Guidelines for Maintenance Hemodialysis in India, 2015.

14 Recepimento della direttiva (UE) 2015/1787 che modifica gli allegati II e III della direttiva 98/83/CE sulla qualità delle acque destinate al consumo umano. Modifica degli allegati II e III del decreto legislativo 2 febbraio 2001, n. 31. (17A05618) (GU Serie Generale n.192 del 18-08-2017).

15 EMA/CHMP/CVMP/QWP/496873/2018 European Medical Agency, 13 November 2018.

16 Water for HEMODIALYSIS and related therapy—Switzerland—UPDATE January 2019.
